# A predictive model involving serum uric acid, C-reactive protein, diabetes, hypercholesteremia, multiple lesions for restenosis risk in everolimus-eluting stent-treated coronary heart disease patients

**DOI:** 10.3389/fcvm.2022.857922

**Published:** 2022-08-11

**Authors:** Qiang Feng, Ying Zhao, Haiyan Wang, Jiayu Zhao, Xun Wang, Jianping Shi

**Affiliations:** ^1^Department of Cardiology, Handan Central Hospital, Handan, China; ^2^Department of Cardiology, The Second Hospital of Hebei Medical University, Shijiazhuang, China

**Keywords:** coronary heart disease, in-stent restenosis, percutaneous coronary intervention, everolimus-eluting stent, predictive factors

## Abstract

**Purpose:**

As a second-generation drug-eluting stent, the restenosis risk factors of the everolimus-eluting stent (EES) lack sufficient evidence. Therefore, the study investigated the in-stent restenosis occurrence and its predictive factors among patients with coronary heart disease (CHD) who underwent percutaneous coronary intervention (PCI) with EES.

**Materials and methods:**

Totally, 235 patients with CHD who underwent PCI with EES were included. At 1 year post PCI with EES (or earlier if clinically indicated), coronary angiography was performed to evaluate the in-stent restenosis status.

**Results:**

Within 1 year post-operation, 20 patients developed in-stent restenosis while 215 patients did not develop in-stent restenosis, resulting in a 1-year in-stent restenosis rate of 8.5%. Diabetes mellitus, hypercholesteremia, hyperuricemia, fasting blood glucose, serum uric acid (SUA), high-sensitivity C-reactive protein (HsCRP), target lesions in the left circumflex artery, patients with two target lesions, length of target lesions and length of stent positively correlated with in-stent restenosis risk, while high-density lipoprotein cholesterol negatively associated with in-stent restenosis risk. Notably, diabetes mellitus, hypercholesteremia, SUA, HsCRP levels, and patients with two target lesions were independent predictive factors for in-stent restenosis risk by multivariate logistic regression analysis. Then, the in-stent restenosis risk prediction model was established based on these independent predictive factors, which exhibited an excellent value in predicting in-stent restenosis risk (area under the curve: 0.863; 95% CI: 0.779–0.848) by receiver operating characteristic analysis.

**Conclusion:**

In-stent restenosis risk prediction model, consisting of diabetes mellitus, hypercholesteremia, SUA, HsCRP, and patients with two target lesions, may predict in-stent restenosis risk in patients with CHD who underwent post-PCI with EES.

## Introduction

Coronary heart disease (CHD), the leading cause of mortality worldwide, refers to the build-up of atherosclerotic plaque in the epicardial coronary arteries, which narrows the coronary artery lumen and impairs the antegrade myocardial blood flow (1, 2). The impaired blood flow eventually results in angina, myocardial infarction, heart failure, arrhythmia, and sudden death (3). Percutaneous coronary intervention (PCI) utilizing stents has been widely adopted as the standard therapy in patients with CHD, and drug eluting-stent is the preferred method in comparison with the traditional bare-metal stents over the last decade (4, 5). Everolimus-eluting stent (EES), the second-generation DES, is introduced with more biocompatible stent polymers than those on first-generation DES [e.g., the sirolimus-eluting stent (SES) and paclitaxel-eluting stent (PES)], which improves arterial healing and decreases the risk of late adverse events (6–10). Nerveless, in-stent restenosis after EES implantation, as the result of arterial damage with subsequent neo-intima hyperplasia, remains the primary clinical problem in treating CHD, which is not negligible (11, 12). Therefore, exploring predictive factors for in-stent restenosis is necessary for guiding the management and improving prognosis in patients with CHD who underwent PCI with EES.

Accumulated studies have illustrated that various clinical and angiographic characteristics, including chronic complications (e.g., diabetes mellitus), abnormal biochemical indexes [e.g., higher serum uric acid (SUA) concentration] and angiographic information (e.g., bifurcation lesions), hold the potential to predict in-stent restenosis risk in patients with CHD who underwent PCI with DES (13–17). While most previously related studies focus on exploring the predictive factors of in-stent restenosis in patients with CHD who underwent PCI with a zotarolimus-eluting stent, PES, or SES, a relevant report regarding EES is limited. Therefore, this study investigated the in-stent restenosis occurrence and its predictive factors in patients with CHD who underwent PCI with EES, aiming to provide insights for better management of in-stent restenosis in these patients with CHD.

## Materials and methods

### Patients

This retrospective study reviewed 235 patients with CHD who underwent PCI with EES in our hospital from January 2016 to December 2018. The patients were eligible for analysis if they had (i) confirmed diagnosis of CHD, (ii) age ≥18 years, (iii) received PCI with EES, (iv) underwent assessment of in-stent restenosis status within 1 year after PCI with EES, (v) medical records and follow-up records were complete (at least included baseline characteristics, operation procedures, and post-procedure management), (vi) no previous PCI, coronary artery bypass grafting, or other cardiovascular major surgery before undergoing PCI with EES, and (vii) no history of malignancies. This study was approved by the Institutional Review Board of our hospital, and written informed consent was collected from all patients or their family members.

### Data collection

By reviewing the medical records, following clinical data of patients were collected: (i) demographic characteristics [such as age, gender, and body mass index (BMI)]; (ii) cardiovascular risk factors [such as current smoke status, hypertension, diabetes mellitus, hypercholesteremia, hyperuricemia, and family history of coronary artery disease (CAD)]; (iii) blood pressure index [mean arterial pressure (MAP)]; (iv) biochemical index [such as fasting blood-glucose (FBG), glycated hemoglobin, triglyceride (TG), total cholesterol (TC), low-density lipoprotein cholesterol (LDL-C), high-density lipoprotein cholesterol (HDL-C), high-sensitivity C-reactive protein (Hs-CRP), erythrocyte sedimentation rate (ESR), white blood cell (WBC), neutrophil, serum creatinine (Scr), and SUA]; (v) cardiac function index [such as left ventricular ejection fraction (LVEF), cardiac troponin I (cTnl), and N-terminal probrain natriuretic peptide (NT-proBNP)]; (vi) angiographic information (such as multivessel artery lesions, location of target lesion, two target lesions, stenosis degree of target lesion and length of target lesion); (vii) operation procedures (such as length of stent, diameter of stent, time of stent dilation and balloon dilation pre stent); (viii) medication used after surgery [such as aspirin, nitrates, statins, β receptor blockers, angiotensin converting enzymes inhibitors (ACEIs), angiotensin receptor blockers (ARBs), and calcium channel blockers].

### In-stent restenosis assessment

The PCI and EES implantation procedures were performed by PCI guidelines (18). Immediately after PCI with EES, coronary angiography was performed for all patients to evaluate the diameter of coronary stenosis. After discharge, if the patients had a clinical indication of in-stent restenosis, coronary angiography was performed to assess the in-stent restenosis status; for the patients without clinical presentation of in-stent restenosis, coronary angiography was required to complete at the 12th month after PCI with EES. The assessment of in-stent restenosis was based on the coronary angiograms by the quantitative coronary angiography (QCA) analysis as previous studies described (19, 20), and the percentage diameter stenosis (PDS) was automatically calculated by the computer-based system Cardiovascular Angiographic Analysis System (CAAS) II (Pie Medical Imaging, Maastricht, Netherlands). The in-stent restenosis was defined as the PDS of the stent-implanted segment at 12th-month follow-up exceeding 50% compared with lumen assessed immediately after PCI with EES (17). Patients were divided into restenosis and non-restenosis groups according to whether they had in-stent restenosis within 1-year follow-up.

### Statistical analysis

Statistical analyses were performed using the SPSS 22 statistical software (SPSS Inc., Chicago, IL, United States), and figure plotting were carried out using the GraphPad Prism 7.01 software (GraphPad Software Inc., San Diego, CA, United States). The continuous data were displayed as mean ± SD, or median and interquartile range (IQR) according to the normality determined by the Kolmogorov–Smirnov test. The categorical data were expressed as count (percentage). Comparison of continuous data between two groups was determined by Student’s *t*-test or the Wilcoxon rank-sum test, and comparison of categorical data between two groups was determined by the chi-square test. Factors predicting in-stent restenosis were analyzed by univariate logistic regression, and the elements with a *P*-value < 0.05 in the univariate logistic regression were further included in the forward stepwise multivariate logistic regression analysis to create the in-stent restenosis risk prediction model and nomogram, whose establishment was referred to the Transparent Reporting of a multivariable prediction model for Individual Prognosis Or Diagnosis (TRIPOD) statement (21). The predicting performance of the in-stent restenosis risk prediction model and each independent predictor for in-stent restenosis was assessed by receiver operating characteristic (ROC) curves and the areas under the curve (AUC) with 95% CIs. *P-*value < 0.05 was considered significant.

## Results

### Clinical features

The mean age of patients with CHD was 63.6 ± 9 years, and there were 192 men/43 women. The mean BMI was 24.7 ± 3.7 kg/m^2^. As for cardiovascular risk factors, 62 (26.4%), 163 (69.4%), 64 (27.2%), 140 (59.6%), 82 (34.9%), and 49 (20.9%) patients with CHD had current smoke, hypertension, diabetes mellitus, hypercholesteremia, hyperuricemia, and family history of CAD, respectively. Regarding cardia function index, the mean LVEF, median cTnl, and median NT-proBNP were 64.5 ± 6.9%, 29.6 (17.4–47) pg/ml, and 75.9 (44.7–125.4) pg/ml, respectively. The detailed information about blood pressure index, biochemical index, angiographic information, operation procedures, and medication used after surgery is shown in Table 1.

**TABLE 1:** Clinical features of CHD patients.

Items	CHD patients (*N* = 235)
Demographic characteristics	
Age (years), mean ± SD	63.6 ± 9.0
Gender (male/female)	192/43
BMI (kg/m^2^), mean ± SD	24.7 ± 3.7
Cardiovascular risk factors	
Current smoke, No. (%)	62 (26.4)
Hypertension, No. (%)	163 (69.4)
Diabetes mellitus, No. (%)	64 (27.2)
Hypercholesteremia, No. (%)	140 (59.6)
Hyperuricemia, No. (%)	82 (34.9)
Family history of CAD, No. (%)	49 (20.9)
Blood pressure index	
MAP (mmHg), mean ± SD	106.0 ± 18.4
Biochemical index	
FBG (mmol/L), mean ± SD	5.9 ± 1.2
Glycated hemoglobin (%), median (IQR)	6.0 (5.0–7.1)
Scr (μmol/L), median (IQR)	82.1 (70.0–92.9)
SUA (μmol/L), median (IQR)	333.0 (280.7–408.9)
TG (mmol/L), median (IQR)	1.8 (0.9–2.5)
TC (mmol/L), mean ± SD	4.5 ± 1.0
LDL-C (mmol/L), mean ± SD	2.7 ± 0.6
HDL-C (mmol/L), mean ± SD	1.0 ± 0.3
HsCRP (mg/L), median (IQR)	4.6 (1.8–8.3)
ESR (mm/L), median (IQR)	12.1 (6.3–20.6)
WBC (×10^9^/L), mean ± SD	6.1 ± 1.5
Neutrophil (×10^9^/L), mean ± SD	3.4 ± 1.0
Cardiac function index	
LVEF (%), mean ± SD	64.5 ± 6.9
cTnI (pg/mL), median (IQR)	29.6 (17.4–47.0)
NT-proBNP (pg/mL), median (IQR)	75.9 (44.7–125.4)
Angiographic information	
Multivessel artery lesions, No. (%)	169 (71.9)
Target lesion at LAD, No. (%)	137 (58.3)
Target lesion at LCX, No. (%)	87 (37.0)
Target lesion at RCA, No. (%)	92 (39.1)
Patients with two target lesions, No. (%)	81 (34.5)
Stenosis degree of target lesion (%), median (IQR)	85.0 (82.0–89.0)
Length of target lesion (mm), median (IQR)	34.0 (26.0–40.0)
Operation procedures	
Length of stent (mm), median (IQR)	37.0 (30.0–43.0)
Diameter of stent (mm), median (IQR)	3.3 (3.0–3.4)
Time of stent dilation (s), median (IQR)	15.0 (13.0–18.0)
Balloon dilation pre stent, No. (%)	73 (31.1)
Medication used after surgery	
Aspirin, No. (%)	235 (100.0)
Nitrates, No. (%)	235 (100.0)
Statins, No. (%)	235 (100.0)
β receptor blockers, No. (%)	204 (86.8)
ACEIs/ARBs, No. (%)	149 (63.4)
Calcium channel blockers, No. (%)	74 (31.5)

CHD, coronary heart disease; BMI, body mass index; CAD, coronary artery disease; MAP, mean arterial pressure; FBG, fasting blood-glucose; Scr, serum creatinine; SUA, serum uric acid; TG, triglyceride; TC, total cholesterol; LDL-C, low density lipoprotein cholesterol; HDL-C, high density lipoprotein cholesterol; HsCRP, high-sensitivity C-reactive protein; ESR, erythrocyte sedimentation rate; WBC, white blood cell; LVEF, left ventricular ejection fraction; cTnI, cardiac troponin I; NT-proBNP, N-terminal-proB-type natriuretic peptide; LAD, left anterior descending branch; LCX, left circumflex artery; RCA, right coronary artery; ACEIs, angiotensin converting enzymes inhibitors; ARBs, angiotensin receptor blockers.

### The occurrence of in-stent restenosis in everolimus-eluting stent-percutaneous coronary intervention treated patients with coronary heart disease

**FIGURE 1:**
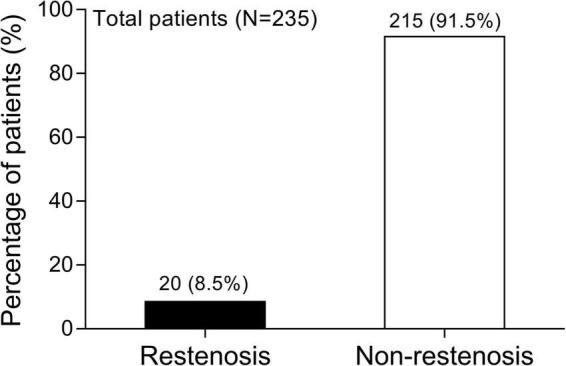
The 1-year in-stent restenosis occurrence. The percentage of patients with CHD who occurred in-stent restenosis and who did not occur in-stent restenosis at 1-year post PCI with EES. CHD, coronary heart disease; PCI, percutaneous coronary intervention; EES, everolimus-eluting stent.

There were 20 patients with CHD who developed in-stent restenosis and 215 patients with CHD who did not develop in-stent restenosis at 1-year post PCI with EES, resulting in a 1-year in-stent restenosis rate of 8.5% (Figure 1).

### Association of clinical features with in-stent restenosis in everolimus-eluting stent-percutaneous coronary intervention treated patients with coronary heart disease

Diabetes mellitus (*P* = 0.017), hypercholesteremia (*P* = 0.015), hyperuricemia (*P* = 0.049), FBG (*P* = 0.036), SUA (*P* = 0.043), HsCRP (*P* < 0.001) levels, target lesion at LCX (*P* = 0.026), patients with two target lesions (*P* = 0.012), length of target lesions (*P* = 0.024), and length of stent (*P* = 0.022) were positively associated with in-stent restenosis risk, while HDL-C (*P* = 0.024) was negatively associated with in-stent restenosis risk in patients with CHD who underwent PCI with EES (Table 2).

**TABLE 2:** Correlation of clinical features with in-stent restenosis.

Items	Restenosis patients (*n* = 20)	Non-restenosis patients (*n* = 215)	*P*-value
Demographic characteristics			
Age (years), mean ± SD	66.7 ± 10.5	63.3 ± 8.9	0.107
Gender, No. (%)			0.316
Female	2 (10.0)	41 (19.1)	
Male	18 (90.0)	174 (80.9)	
BMI (kg/m^2^), mean ± SD	25.2 ± 3.2	24.6 ± 3.8	0.488
Cardiovascular risk factors			
Current smoke, No. (%)	7 (35.0)	55 (25.6)	0.361
Hypertension, No. (%)	17 (85.0)	146 (67.9)	0.113
Diabetes mellitus, No. (%)	10 (50.0)	54 (25.1)	**0.017**
Hypercholesteremia, No. (%)	17 (85.0)	123 (57.2)	**0.015**
Hyperuricemia, No. (%)	11 (55.0)	71 (33.0)	**0.049**
Family history of CAD, No. (%)	6 (30.0)	43 (20.0)	0.292
Blood pressure index			
MAP (mmHg), mean ± SD	107.2 ± 14.4	105.9 ± 18.7	0.779
Biochemical index			
FBG (mmol/L), mean ± SD	6.4 ± 1.0	5.8 ± 1.2	**0.036**
Glycated hemoglobin (%), median (IQR)	5.4 (5.0–7.5)	6.1 (4.9–7.1)	0.799
Scr (μmol/L), median (IQR)	87.3 (71.6–96.2)	82.0 (70.0–92.6)	0.259
SUA (μmol/L), median (IQR)	418.6 (287.8–483.8)	332.7 (280.4–400.1)	**0.043**
TG (mmol/L), median (IQR)	1.7 (1.0–2.9)	1.8 (0.9–2.5)	0.544
TC (mmol/L), mean ± SD	4.6 ± 0.8	4.5 ± 1.0	0.919
LDL-C (mmol/L), mean ± SD	2.9 ± 0.6	2.7 ± 0.6	0.131
HDL-C (mmol/L), mean ± SD	0.9 ± 0.2	1.0 ± 0.3	**0.024**
HsCRP (mg/L), median (IQR)	7.4 (5.9–10.1)	4.1 (1.5–8.0)	**<0.001**
ESR (mm/L), median (IQR)	14.2 (8.6–25.2)	12.0 (5.2–20.4)	0.195
WBC (×10^9^/L), mean ± SD	6.4 ± 1.4	6.1 ± 1.5	0.348
Neutrophil (10^9^/L), mean ± SD	3.6 ± 1.2	3.4 ± 0.9	0.256
Cardiac function index			
LVEF (%), mean ± SD	62.4 ± 6.8	64.7 ± 6.8	0.156
cTnI (pg/mL), median (IQR)	36.4 (23.2–50.7)	29.6 (16.6–46.6)	0.126
NT-proBNP (pg/mL), median (IQR)	76.9 (58.3–145.0)	75.9 (42.4–124.2)	0.299
Angiographic information			
Multivessel artery lesions, No. (%)	18 (90.0)	151 (70.2)	0.060
Target lesion at LAD, No. (%)	13 (65.0)	124 (57.7)	0.525
Target lesion at LCX, No. (%)	12 (60.0)	75 (34.9)	**0.026**
Target lesion at RCA, No. (%)	7 (35.0)	85 (39.5)	0.691
Patients with two target lesions, No. (%)	12 (60.0)	69 (32.1)	**0.012**
Stenosis degree of target lesion (%), median (IQR)	85.5 (83.0–91.0)	85.0 (82.0–88.0)	0.258
Length of target lesion (mm), median (IQR)	39.0 (29.0–46.0)	33.0 (26.0–39.0)	**0.024**
Operation procedures			
Length of stent (mm), median (IQR)	42.5 (34.0–49.0)	37.0 (29.0–43.0)	**0.022**
Diameter of stent (mm), median (IQR)	3.1 (3.0–3.3)	3.3 (3.0–3.4)	0.420
Time of stent dilation (s), median (IQR)	15.0 (12.0–19.8)	15.0 (13.0–18.0)	0.893
Balloon dilation pre stent, No. (%)			
Medication used after surgery			
Aspirin, No. (%)	20 (100.0)	215 (100.0)	–
Nitrates, No. (%)	20 (100.0)	215 (100.0)	–
Statins, No. (%)	20 (100.0)	215 (100.0)	–
β receptor blockers, No. (%)	16 (80.0)	188 (87.4)	0.347
ACEIs/ARBs, No. (%)	10 (50.0)	139 (64.7)	0.193
Calcium channel blockers, No. (%)	6 (30.0)	68 (31.6)	0.881

Comparison was determined by Student’s t test, Wilcoxon rank sum test or Chi-square test. Boldface represented P value < 0.05. SD, standard deviation; BMI, body mass index; CAD, coronary artery disease; MAP, mean arterial pressure; FBG, fasting blood-glucose; IQR, interquartile range; Scr, serum creatinine; SUA, serum uric acid; TG, triglyceride; TC, total cholesterol; LDL-C, low density lipoprotein cholesterol; HDL-C, high density lipoprotein cholesterol; HsCRP, high-sensitivity C-reactive protein; ESR, erythrocyte sedimentation rate; WBC, white blood cell; LVEF, left ventricular ejection fraction; cTnI, cardiac troponin I; NT-proBNP, N-terminal-proB-type natriuretic peptide; LAD, left anterior descending branch; LCX, left circumflex artery; RCA, right coronary artery; ACEIs, angiotensin converting enzymes inhibitors; ARBs, angiotensin receptor blockers.

### Analysis of factors predicting in-stent restenosis in everolimus-eluting stent-percutaneous coronary intervention treated patients with coronary heart disease

Univariate logistic regression analysis displayed that diabetes mellitus (*P* = 0.021; OR = 2.981), hypercholesteremia (*P* = 0.024; OR = 4.238), FBG (*P* = 0.037; OR = 1.489), SUA (*P* = 0.014; OR = 1.008), HsCRP (*P* = 0.004; OR = 1.152), target lesion at LCX (*P* = 0.031; OR = 2.8), patients with two target lesions (*P* = 0.016; OR = 3.174), length of target lesion (*P* = 0.02; OR = 1.064), and length of stent (*P* = 0.023; OR = 1.062) correlated with inclined in-stent restenosis risk, while HDL-C level (*P* = 0.027; OR = 0.092) correlated with declined in-stent restenosis risk in patients with CHD who underwent PCI with EES (Table 3).

**TABLE 3:** Factors predicting in-stent restenosis.

Items	Univariate logistic regression model			
	*P*-value	OR	95% CI	
			Lower	Higher
Age	0.111	1.043	0.990	1.098
Male	0.326	2.121	0.473	9.504
BMI	0.486	1.043	0.927	1.174
Current smoke	0.364	1.566	0.595	4.126
Hypertension	0.126	2.678	0.759	9.445
Diabetes mellitus	**0.021**	2.981	1.177	7.550
Hypercholesteremia	**0.024**	4.238	1.206	14.894
Hyperuricemia	0.055	2.479	0.982	6.255
Family history of CAD	0.297	1.714	0.622	4.721
MAP	0.778	1.004	0.979	1.029
FBG	**0.037**	1.489	1.024	2.164
Glycated hemoglobin	0.927	1.012	0.788	1.299
Scr	0.109	1.023	0.995	1.052
SUA	**0.014**	1.008	1.002	1.014
TG	0.634	1.129	0.685	1.862
TC	0.919	1.024	0.644	1.631
LDL-C	0.133	1.787	0.839	3.808
HDL-C	**0.027**	0.092	0.011	0.758
HsCRP	**0.004**	1.152	1.047	1.268
ESR	0.262	1.027	0.980	1.076
WBC	0.347	1.157	0.854	1.566
Neutrophil	0.256	1.324	0.816	2.148
LVEF	0.158	0.950	0.884	1.020
cTnI	0.261	1.012	0.991	1.033
NT-proBNP	0.404	1.003	0.996	1.010
Multivessel artery lesions	0.078	3.815	0.860	16.923
Target lesion at LAD	0.526	1.363	0.523	3.552
Target lesion at LCX	**0.031**	2.800	1.096	7.150
Target lesion at RCA	0.691	0.824	0.316	2.148
Patients with two target lesions	**0.016**	3.174	1.241	8.119
Stenosis degree of target lesion	0.199	1.068	0.966	1.181
Length of target lesion	**0.020**	1.064	1.010	1.122
Length of stent	**0.023**	1.062	1.009	1.118
Diameter of stent	0.686	0.755	0.194	2.944
Time of stent dilation	0.753	0.981	0.873	1.103
Balloon dilation pre stent	0.914	0.947	0.349	2.571
β receptor blockers	0.352	0.574	0.179	1.846
ACEIs/ARBs	0.198	0.547	0.218	1.372
Calcium channel blockers	0.881	0.926	0.341	2.515

Factors predicting in-stent restenosis were analyzed by univariate logistic regression model. Boldface represented P value < 0.05. OR, odds ratio; CI, confidence interval; BMI, body mass index; CAD, coronary artery disease; MAP, mean arterial pressure; FBG, fasting blood-glucose; Scr, serum creatinine; SUA, serum uric acid; TG, triglyceride; TC, total cholesterol; LDL-C, low density lipoprotein cholesterol; HDL-C, high density lipoprotein cholesterol; HsCRP, high-sensitivity C-reactive protein; ESR, erythrocyte sedimentation rate; WBC, white blood cell; LVEF, left ventricular ejection fraction; cTnI, cardiac troponin I; NT-proBNP, N-terminal-proB-type natriuretic peptide; LAD, left anterior descending branch; LCX, left circumflex artery; RCA, right coronary artery; ACEIs, angiotensin converting enzymes inhibitors; ARBs, angiotensin receptor blockers.

### Analysis of factors independently predicting in-stent stenosis in everolimus-eluting stent-percutaneous coronary intervention treated patients with coronary heart disease

**FIGURE 2:**
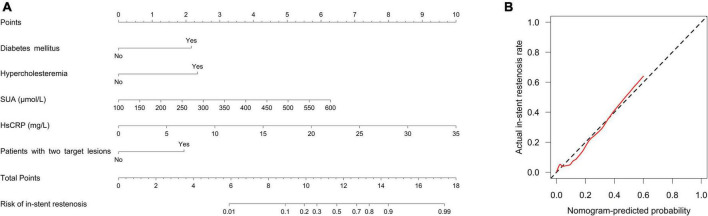
A nomogram for in-stent restenosis risk. The proposed nomogram **(A)** and the calibration plot of the proposed nomogram **(B)** for in-stent restenosis risk. The red line indicates degree of fitting and the black dashed line indicates calibration curve.

Forward stepwise multivariate logistic regression analysis disclosed that diabetes mellitus (*P* = 0.003; OR = 5.63), hypercholesteremia (*P* = 0.013; OR = 6.514), SUA (*P* = 0.004; OR = 1.01), HsCRP (*P* < 0.001; OR = 1.257), and patients with two target lesions (*P* = 0.005; OR = 4.731) were independent predictive factors for higher in-stent stenosis risk in patients with CHD who underwent PCI with EES (Table 4). Subsequently, these independent factors were used to develop an in-stent restenosis risk estimation nomogram (Figure 2A), and the calibration plots disclosed good consistency between the observed probabilities and the nomogram’s predictions regarding the in-stent restenosis risk (Figure 2B).

**TABLE 4:** Factors independently predicting in-stent restenosis.

Items	Forward stepwise multivariate logistic regression model			
	*P*-value	OR	95% CI	
			Lower	Higher
Diabetes mellitus	0.003	5.630	1.766	17.941
Hypercholesteremia	0.013	6.514	1.496	28.363
SUA	0.004	1.010	1.003	1.017
HsCRP	<0.001	1.257	1.123	1.406
Patients with two target lesions	0.005	4.731	1.589	14.084

Factors with *P* value < 0.05 in univariate logistic regression model were included in this forward stepwise multivariate logistic regression model to screen independent predictors. The predictive model of in-stent restenosis was as follows: *P* = e^ [−10.322 + 1.728 (diabetes mellitus) + 1.874 (hypercholesteremia) + 0.010 (SUA) + 0.229 (HsCRP) + 1.554 (patients with two target lesions)] / 1 + e^ [−10.322 + 1.728 (diabetes mellitus) + 1.874 (hypercholesteremia) + 0.010 (SUA) + 0.229 (HsCRP) + 1.554 (patients with two target lesions)], −2Ln(L) = 95.594. OR, odds ratio; CI, confidence interval; SUA, serum uric acid; HsCRP, high-sensitivity C-reactive protein.

### Receiver operating characteristic curve analysis

**FIGURE 3:**
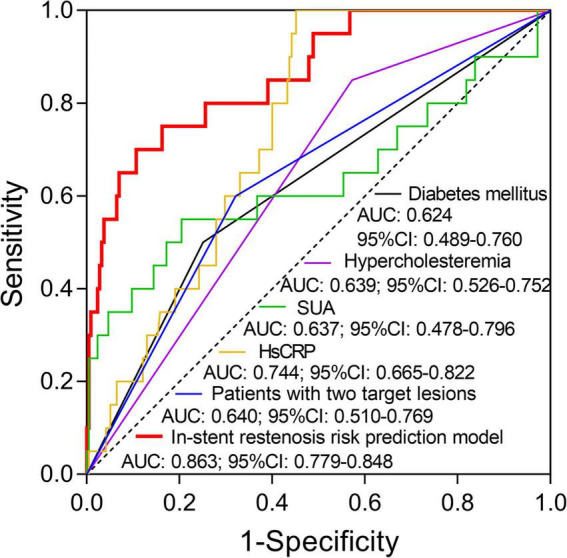
The value of the in-stent restenosis risk prediction model and each independent predictive factor for in-stent restenosis risk. The performance of diabetes mellitus, hypercholesteremia, SUA, HsCRP, patients with two target lesions, and in-stent restenosis risk prediction model in predicting in-stent restenosis risk in patients with CHD who underwent post-PCI with EES. SUA, serum uric acid; HsCRP, high-sensitivity C-reactive protein; CHD, coronary heart disease; PCI, percutaneous coronary intervention; EES, everolimus-eluting stent.

Independent predictive factors for in-stent stenosis risk were used to create in-stent restenosis risk prediction model by forward stepwise multivariate logistic regression analysis and the formula was as follow: *P* = e^ [−10.322 + 1.728 (diabetes mellitus) + 1.874 (hypercholesteremia) + 0.01 (SUA) + 0.229 (HsCRP) + 1.554 (patients with two target lesions)]/1 + e^ [−10.322 + 1.728 (diabetes mellitus) + 1.874 (hypercholesteremia) + 0.01 (SUA) + 0.229 (HsCRP) + 1.554 (patients with two target lesions)], −2Ln(L) = 95.594. Subsequently, the ability of in-stent restenosis risk prediction model and each independent predictive factor was analyzed by ROC curve analysis. It was revealed that hypercholesteremia (AUC: 0.639; 95% CI: 0.526–0.752), HsCRP (AUC: 0.744; 95% CI: 0.665–0.822) and patients with two target lesions (AUC: 0.64; 95% CI: 0.51–0.769) could predict in-stent restenosis risk, while diabetes mellitus (AUC: 0.624; 95% CI: 0.489–0.76) and SUA (AUC: 0.637; 95% CI: 0.478–0.796) could predict in-stent restenosis risk to a certain extent in patients with CHD who underwent PCI with EES (Figure 3). In terms of the in-stent restenosis risk prediction model, it exhibited a good predictive value for in-stent restenosis risk in patients with CHD who underwent PCI with EES (AUC: 0.863; 95% CI: 0.779–0.848).

## Discussion

The current study mainly observed that the 1-year in-stent restenosis rate of EES was 8.5%, meanwhile, diabetes mellitus, hypercholesteremia, SUA, HsCRP, and two target lesions were independent factors for increased restenosis risk, their combination showed a good predictive value for in-stent restenosis risk with AUC of 0.863.

The introduction of EES represents a great leap forward in decreasing in-stent restenosis risk and target lesion revascularization rates after PCI (22). Nerveless EES is not immune to in-stent restenosis, and in-stent restenosis still occurs in 3–9.2% of patients who underwent PCI with EES (23–28). A study reports an 18-month restenosis rate of 9.2% in patients with left central coronary artery disease after EES implantation (24). Another study illustrates that 8.7% of hemodialysis patients occurred restenosis at an 8-month follow-up after receiving EES for coronary intervention (25). This study observed that the 1-year in-stent restenosis rate was 8.5% in patients with CHD after PCI with EES, which was within the range of the reported in-stent restenosis occurrence in patients who underwent PCI with EES by the previous studies. The slight difference existing among studies might result from different follow-up duration (12 months) and different study populations (patients with CHD).

Previous works of research have illuminated the potential factors associated with in-stent restenosis risk in patients with CHD after PCI with a zotarolimus-eluting stent, PES, or SES (13–17). Increased hypertension prevalence, diabetes mellitus prevalence, higher SUA, LDL-C, Hs-CRP concentrations, more target lesions, and longer length of stent correlated with raised restenosis risk in patients with CHD after PCI with PES or SES (17). Another study discloses that longer lesion length and more in-stent restenotic lesions are independent predictive factors for elevated restenosis risk in patients who underwent PCI with SES (16). The predictive factors for in-stent restenosis risk in patients with CHD after PCI with EES are not reported yet. This study revealed that diabetes mellitus, hypercholesteremia, hyperuricemia, FBG, SUA, HsCRP, more target lesions at LCX, patients with two target lesions, length of the target lesion, and length of stent were predictive factors for higher in-stent restenosis risk. At the same time, HDL-C level was a predictive factor for lower in-stent restenosis risk in patients with CHD who underwent PCI with EES.

Recently, several studies have established several models for predicting in-stent restenosis in patients undergoing percutaneous coronary intervention and those patients with triple-vessel disease after second-generation drug-eluting stent implantation (29–31). For instance, one study shows that a prediction nomogram which includes the prior PCI, hyperglycemia, stents in the left anterior descending artery, stent type, and absence of clopidogrel has a good ability in predicting the in-stent restenosis in patients undergoing percutaneous coronary intervention (29). Another study discloses that after adjustment by multivariate logistic regression analyses older age, current smoking, and CKD4-5 are considered independent risk factors for in-stent restenosis in triple-vessel disease after second-generation drug-eluting stent implantation (30). Additionally, this study disclosed that diabetes mellitus, hypercholesteremia, SUA, HsCRP, and patients with two target lesions were independent predictive factors for increased restenosis risk in patients with CHD who underwent PCI with EES by multivariate logistic regression analysis. Then, the in-stent restenosis risk prediction model was constructed based on these independent predictive factors, and a ROC curve analysis was conducted. It was revealed that this prediction model exhibited a good value in predicting inclined in-stent restenosis risk in patients with CHD who underwent PCI with EES (AUC: 0.863; 95% CI: 0.779–0.848), which might help with the management of in-stent restenosis in patients with CHD who underwent post-PCI with EES in clinical practice.

Several limitations of this study need to be stated. Firstly, sample size (*N* = 235) was a major limitation, and a multi-center registry including a large number of patients could allow more supported conclusions. Secondly, patients with CHD were only followed up for a relatively short period (12 months), thus correlation of candidate factors with long-term restenosis risk needed further investigation. Thirdly, as this study was a retrospective study, thereby further prospective study is needed to validate our findings.

In conclusion, the in-stent restenosis risk prediction model, consisting of diabetes mellitus, hypercholesteremia, SUA, HsCRP, and patients with two target lesions, exhibits the potential as a good marker for in-stent restenosis risk in patients with CHD who underwent PCI with EES.

## Data availability statement

The original contributions presented in this study are included in the article/supplementary material, further inquiries can be directed to the corresponding author.

## Ethics statement

The studies involving human participants were reviewed and approved by the Handan Central Hospital. The patients/participants provided their written informed consent to participate in this study.

## Author contributions

QF and HW contributed to conception and design and revised the article critically for important intellectual content. QF, HW, and JS contributed to the acquisition of data. QF, YZ, JZ, and XW contributed to the analysis of data. All authors drafted and revised the article and approved the final version to be published.
